# Public health policy and political contestation in Indonesia

**DOI:** 10.4102/jphia.v15i1.646

**Published:** 2024-07-25

**Authors:** Cashtri Meher, Fotarisman Zaluchu

**Affiliations:** 1 Faculty of Medicine, Universitas Islam Sumatera Utara, Medan, Indonesia; 2 Department of Social Anthropology, Faculty of Social Science and Political Science, Universitas Sumatera Utara, Medan, Indonesia

**Keywords:** public health, political campaign, election, Indonesia, stunting, women’s health

## Abstract

Public health issues should be a focal concern for public leaders. A critical moment for articulating intended policies is during elections. At this time, candidates present significant ideas and proposals derived from the evaluation and reflection on the previous administration’s governance. This approach ensures that the proposed programmes are grounded in evidence. In 2024, Indonesia conducted general elections, amid significant public health challenges such as the persistently high prevalence of stunting and poor maternal and child health outcomes. The Prabowo-Gibran, who then won the election, focused their campaign on providing free food and milk. This campaign appears to be unsupported by a comprehensive improvement plan, leading to the impression that public health issues are merely used to enhance electoral appeal.

## Introduction

In the contemporary political landscape, health programmes respond to community needs and are not only seen as strategic tools in electoral campaigns. In Indonesia, in 2024, a national competition involving three pairs of candidates took place. One of the pairs that eventually won the competition was Prabowo Subianto and Gibran Rakabumi Raka (abbreviated as Prabowo-Gibran). This pair received a very significant number of votes. One of their campaign content that became trending was efforts to reduce stunting and improve maternal and child health. During the national campaign, Prabowo-Gibran promised free lunch and milk at schools and nutritional assistance for toddlers and pregnant women. The lunch and milk programme targeted thousands of recipients every week and would reach around 80 million by the end of their administration. Whether liked or not, the ‘free’ icon attracted public attention, significantly increasing their support base. The pair, declared just hours before the registration closed, must be acknowledged for spreading a campaign liked by the community.

The purpose of this opinion is to re-voice populist health initiatives based on the general election results by taking the Prabowo-Gibran campaign as a case study and comparing it with health programmes implemented so far. A critical examination will highlight the implementation of programmes similar to those of the following government.

## Nutritional issues

Based on the data from the Indonesia Nutritional Status Survey (SSGI) 2022 and 2023, the prevalence of stunting in Indonesia does show a decreasing trend. However, it is still a serious public health problem. According to SSGI 2022, the prevalence of stunting is about 24.4% among children under 5 years old. This figure marks a decrease compared to previous years, but still indicates that about one in four children in Indonesia experiences stunting. Although, according to SSGI 2023,^1^ the prevalence of stunting dropped to about 21.7%, this indicator does not disregard the continuity of the stunting problem in those who previously suffered from this syndrome.

When viewed by region, there is also a striking disparity, reflecting differences in socio-economic conditions, access to health services and the availability of nutritious food. On the island of Java, although generally having a lower prevalence than other regions in Indonesia, some areas in Central and East Java still record relatively high figures. Meanwhile, although there is a decrease in the Sumatra region, stunting prevalence is still higher than the national average, especially in provinces such as Aceh and North Sumatra. In Kalimantan, some areas still have a high prevalence of stunting, especially in more isolated rural areas. Meanwhile, Sulawesi and Papua are the most affected regions, with some provinces recording very high stunting prevalence, reflecting significant challenges in access to resources and health services.

## Prevention

Providing daily food along with milk and other nutritious foods is good. Unfortunately, that is not prevention. Take stunting, for example. It is typical for toddlers to experience stunting from the time they are born until they are 5 years old. If food intake only begins after birth, it is too late. Their brain development has already occurred because the growth process of the essential organ in life had already begun from the first day the foetus was formed.

Similarly, the issue of malnutrition is very much related to the condition of the baby while in the womb, not after birth. From within the womb, the proteins composing the baby’s internal organs work to prepare the basis for the baby’s future organs development like lung and heart. After birth, it is just about perfecting it.

This condition is not much different from the health of pregnant and nursing mothers. The low health status of pregnant mothers that then contributes to maternal deaths during childbirth and 40 days afterward is not only determined by what they eat during pregnancy and breastfeeding, as will be provided by the Prabowo-Gibran government. However, the mother’s health is determined by various factors that vary with each mother before pregnancy, including marital age, the mother’s position in the family and other social factors.^2,3,4,5,6^

Thus, addressing issues after a child is born or when a mother is already pregnant is very ineffective. Every incidence of stunting, malnutrition and health status that is not prevented upstream will become new cases that will continuously arise and become new problems. If left unaddressed, Indonesia will never solve the real problem. The free food programme, whether for toddlers, school-aged children or pregnant women, only targets existing problems, not preventing similar events.

The authors even suspect that this programme will shift from the intended beneficiaries. The authors have evaluated the provision of supplementary food intended for school-aged children. These food items should ideally be sourced from local products. Ideally, that is how it should be. However, its preparation involved food products that only purchase ready-made ones. Not only that, but the food products given to the beneficiaries also do not comply with the recommended nutritional value guidelines. As a result, on paper, the supplementary food programme has a high coverage. However, its contribution to other indicators, such as education quality and the health status of school-aged children, is not optimal.

What may be more shocking is the provision of high-protein biscuits to pregnant women. This programme is an innovation from the Ministry of Health to prevent stunting during pregnancy. In reality, the mothers who were supposed to consume these biscuits during months of pregnancy eventually got bored with the taste of the biscuits they continuously received. So, they only took their biscuit ration but gave it to their children or husbands to eat.

## Electoral strategy

The Prabowo-Gibran programme is a highly intriguing plan. However, based on experience, the realities previously encountered should serve as a reflection. Scientific-based evaluation should be the basis for developing health programme concepts.^7^ Using evidence-based evaluation, the health programmes to be delivered undergo evaluation and improvement.

Unfortunately, what is happening is the use of public health issues only as a mere electoral political strategy. Public choice theory, often used to analyse political behaviour in the context of public policy, states that political leaders promote policies they believe will garner the most votes. The basic assumption of this theory is not much different from the principles of marketing, where maximum efforts are made to achieve profit. With this assumption, populist health programmes are designed to be an electoral strategy expected to attract votes from specific demographics, especially groups that will most directly benefit from the policies.

In Indonesia, more than half of the voters are female. They typically have to work to meet their family’s needs, including lunch and milk for their children and themselves if pregnant. To some extent, this campaign targets them, increasing the electability of the Prabowo-Gibran pair. In the eyes of these voters, the campaign of lunch and milk wrapped with ‘free’ is an effort to improve family nutrition. This programme has even entered into the strategy of the first 100 days of the government that will be inaugurated in October 2024.

In addition to the group of women, the target voter group is people experiencing poverty. As many as 25 million Indonesians still belong to the poor group who are naturally very interested in becoming beneficiaries of this campaign.^8^ That means as many as 9.36% of the population who cannot meet their fundamental rights directly or indirectly want to pin their hopes on Prabowo-Gibran.

However, we offer a view for improving the success of the Prabowo-Gibran programme. Three things will be the keywords. Firstly, the extent to which community participation and support is provided. The success of health programmes in a country as large as Indonesia, which has many problems, often depends on how well the target community receives the programme. Local participation in planning and execution can enhance acceptance and effectiveness (see, for instance, Zaluchu^9^). Secondly, funding. On paper, this programme has a trillion Indonesian Rupiahs (IDR) figure to guarantee its implementation. Successful programmes are usually supported by stable and sustainable funding that allows long-term implementation without disrupting public finances. Government debt is a susceptible issue, given Indonesia’s increasingly worrying public debt ratio. As this programme requires substantial funding, the new government needs to involve the private sector for contributions. Government funds are insufficient, thus public financing can also be considered as an option. And thirdly, transparency and accountability. Programmes with robust monitoring and evaluation mechanisms tend to be more successful because the potential for misuse of funds and resources is smaller. In contrast, Indonesia is one of the countries with a Corruption Perception Index that is still poor in the region. Therefore, the government must involve external parties, including non-government organisations (NGOs), to oversee the implementation of this programme in order to prevent the misuse of state finances.

Populist health programmes provide a concrete example of how political leaders use public policy to secure electoral support. The Prabowo-Gibran case shows that promises targeting basic needs such as health can play a significant role in electoral strategy. The victory of the Prabowo-Gibran pair is an important experiment to serve as a reflection for health policy experts.
